# Congenital Limb Deformities in a Neonatal Crossbred Pig

**DOI:** 10.1155/2022/5516633

**Published:** 2022-04-26

**Authors:** Michael C. Rahe, Laurence Evrard, Neil B. Holmes

**Affiliations:** ^1^Department of Veterinary Diagnostic and Production Animal Medicine, College of Veterinary Medicine, Iowa State University, Ames, IA, USA; ^2^VetCT, St John's Innovation Centre, Cowley Road, Cambridge CB4 0WS, UK; ^3^Gila River Veterinary Services, LLC, 1868 S 232nd Ln, Buckeye, AZ 85326, USA

## Abstract

**Purpose:**

To describe the pathology and imaging findings in two neonatal piglets with congenital limb deformities.

**Methods:**

The litter from a second parity crossbred sow presented with four mummified fetuses, three stillborn piglets, and two live piglets with notable limb deformities that were unable to effectively ambulate. The piglets were euthanized and submitted for gross and histological evaluation.

**Results:**

Both pigs had bilateral secondary cleft palates, with hypoplasia of the nasal turbinates, and external rotation of the forelimbs. One pig displayed bilateral cryptorchidism, markedly thin and shortened hindlimbs, and syndactyly of both hind feet. Radiographs and gross dissection confirmed the presence of single ossified proximal to distal phalanges of both feet, bilaterally shortened tibias with fibular aplasia, and delayed ossification of tarsal as well as carpal bones.

**Conclusions:**

To the author's knowledge, this is the first reported case of hindlimb meromelia with syndactyly in a pig.

## 1. Introduction

Skeletal dysplasia is a common finding in veterinary medicine which is characterized by localized anomalies of the developing skeleton. This is most frequently encountered with chondrodystrophic breeds of dogs and cats. However, there are also rare examples of skeletal dysplasia reported in production animals and wildlife, such as syndactyly, polymelia, and hemimelia (1–3). These anomalies are often incompatible with limb function and are frequently caused by genetic disorders, although other differentials (toxicities, viruses, etc.) which can result in the impairment of skeletal development need also be considered. The rarity of these cases requires their description in the published literature so that potential causes may be investigated and identified. Here, we present a case of congenital limb deformities affecting two piglets from the litter of a second parity crossbred sow.

## 2. Case Presentation

A second parity crossbred sow farrowed a litter composed of four mummified fetuses, three stillborn piglets, and two grossly abnormal live piglets that were unable to effectively ambulate. Due to the piglets' inability to move when the sow laid down or walk to the teat in order to nurse, the piglets were euthanized and submitted to the Iowa State University diagnostic laboratory to rule out potential infectious agents that could affect the other sows on the farm.

**Figure 1:**
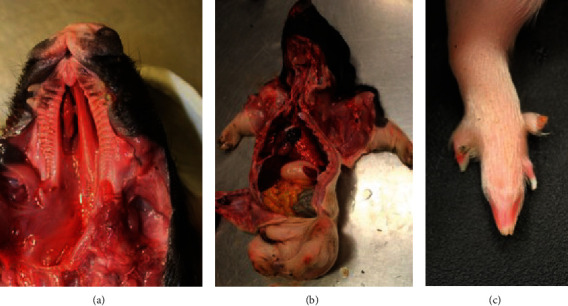
Neonatal pig #1. (a) Bilateral cleft palate with hypoplasia of nasal turbinates. (b) Bilaterally shortened hindlimbs. (c) Syndactyly with dewclaws and vestigial nonossified keratin structure.

Evaluation of the limbs and gross necropsy revealed that both piglets had externally rotated forelimbs and cleft palates with nasal turbinate hypoplasia (Figure 1(a)). Pig #1 displayed bilateral cryptorchidism and very thin and shortened hindlimbs ending in single central digits or syndactyly (Figures 1(b) and 1(c)). The dewclaws were intact. The rest of the thoracic and abdominal organs, spinal columns, and the brains from both pigs were grossly unremarkable.

**Figure 2:**
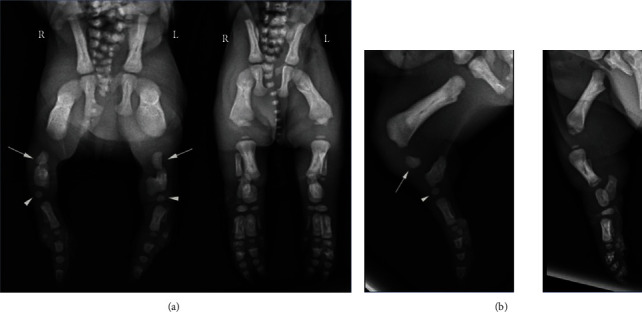
Comparative radiographs of pig #1 with syndactyly (left) and pig #2 with normal findings (right) illustrate the single digit fully developed in pig #1. Note also the markedly shortened tibias with absent fibulas (arrows) and underdeveloped tarsal cuboid bones (arrowheads): (a) craniocaudal projection; (b) mediolateral projections.

Utilizing a Summit HF30 X-ray machine, craniocaudal and mediolateral radiographs of the forelimbs and hindlimbs of pig #2, with grossly unremarkable hindlimbs, revealed normal findings of the appendicular skeleton. However, radiographs, of the same orientation, of pig #1, with shortened hindlimbs and syndactyly, showed normal forelimbs but bilateral hindlimb meromelia, defined as partial absence of a free limb (Figure 2). This was both terminal and transverse. Transverse in that both the fibula (aplasia) and tibia (shortened) were affected and terminal in that there were rudimentary tarsal cubit bones ending distally in the complete fusion of bone between the two central digital rays, which is also compatible with a diagnosis of complex syndactyly. There was also delayed ossification of carpal and tarsal bones. Histopathologic evaluation, utilizing an Olympus BX41 brightfield microscope, of haematoxylin and eosin stained slides of the tibial bones and phalanges revealed no abnormalities in the microscopic composition of the bone nor in endochondral ossification.

## 3. Discussion

Tibial hemimelia is a very rare congenital defect described in several mammalian species including humans and certain lines of cattle. The anomaly is characterized by a poorly developed and subsequently shortened tibia and can occur by itself or be part of complex malformation syndromes, which can include but are not limited to the following congenital defects: syndactyly, polydactyly, cleft palate, abdominal hernia, meningocele, and cryptorchidism (4–6). Previous research, in both humans and cattle, has found this condition to have an autosomal recessive inheritance (4, 7). In these species, mutations in several genes, which are important for mammalian skull, limb, and digit development, most notably Hoxd-12, Aristaless-like homeobox 4 (ALX4), zinc finger protein GLI3, and sonic hedgehog (SHH), have been reported with the development of tibial hemimelia (1, 8, 9).

The presented case shares many similarities with previously described cases of tibial hemimelia from other species, including the bilaterally shortened tibial bones and malformations in additional tissues. However, rather than naming this condition, tibial hemimelia, the Frantz-O'Rahilly classification system for nomenclature of congenital skeletal limb deficiencies, which is used for child limb deformities in humans, was utilized due to its specific means of describing congenital skeletal abnormalities. Currently, an appropriate nomenclature system for congenital skeletal limb deficiencies in veterinary animals does not exist, and this report is meant to as specifically as possible describe what was observed. The Frantz-O'Rahilly system defines partial absence of a free limb as meromelia and eschews several clinical terms, such as hemimelia and peromelia, in favour of describing the limb abnormality from general to specific: amelia or peromelia, followed by basic headings of terminal or intercalary and subgroups of transverse and longitudinal as well as citation of the absence or malformation of specific bones. This was described as a shortened limb (peromelia), which is terminal in that the defects extended distally through the phalanges. It is also considered transverse as both the tibia and fibula were affected. While most of veterinary medicine still employs clinical descriptive terms (e.g., hemimelia) to describe partial absence of a limb, the adoption of a uniform system allowing for specific identification of affected bones would result in a more accurate description of findings.

There is no clear cause for the described congenital abnormalities. While an autosomal recessive genetic mutation is possible, this second parity sow's previous litter was sired by the same boar that sired her completely normal first litter. This boar had not previously been associated with congenital anomalies in other litters that it had sired. Furthermore, this is the first report of these congenital defects being described on this farm, and there is no known close genetic lineage between the boar and sow. Chromosomal instability is another differential for the noted congenital limb abnormalities and has been previously described in hindering limb development in calves and sheep (10, 11). The cause of chromosomal instability is not always known, although mutagenic compounds, ionizing radiation, and genetic disorders have been reported (12, 13). In this case, sows are housed indoors with no known exposure to DNA damaging compounds or radiation. Additionally, they are fed a diet free of potential teratogenic plants, such as poison hemlock (*Conium maculatum*), tobacco (*Nicotiana tabacum*), and lupine (*Lupinus* spp.) (14). Common causes of porcine abortion and fetal mummification were tested for. PCRs on neonatal tissue for porcine reproductive and respiratory virus (PRRSV), porcine parvovirus-1 (PPV-1), and porcine circovirus 2 (PCV2) were all negative, and there was no evidence of viral infection on histopathologic evaluation of tissue.

In conclusion, the described limb malformations, coupled with additional congenital abnormalities in this pig, present a complex of malformations similar to those described in cattle and humans which are linked to specific genetic conditions. Therefore, it is likely that the presented case also has a genetic basis; however, inheritance pattern was not able to be pursued due to small sample size. In future, if similar abnormalities in swine are observed, analysis of lineage and candidate genes previously associated with tibial hemimelia in cattle and humans is advisable for attempts to find a genetic cause for these defects.

## Data Availability

The authors are open to sharing samples for genetic testing, which are stored in long term -80°C storage at the Iowa State University Veterinary Diagnostic Laboratory.
